# Aberrant Expression of CYP2W1 in Pediatric Soft Tissue Sarcomas: Clinical Significance and Potential as a Therapeutic Target

**DOI:** 10.3390/curroncol32030131

**Published:** 2025-02-26

**Authors:** Dora Molina-Ortiz, Carmen Torres-Zárate, Rocío Cárdenas-Cardós, Daniel Hernández-Arrazola, Marco R. Aguilar-Ortiz, José Palacios-Acosta, Jaime Shalkow-Klincovstein, Víctor Dorado-González, Rebeca Santes-Palacios, Elizabeth Hernández-Urzúa, Araceli Vences-Mejía

**Affiliations:** 1 Laboratory of Genetic Toxicology, National Institute of Pediatrics, Mexico City 04530, Mexico; doramolina29@yahoo.com.mx (D.M.-O.); araceli_488@hotmail.com (C.T.-Z.); vmdgod@yahoo.com.mx (V.D.-G.); rsantesp@pediatria.gob.mx (R.S.-P.); elyzabet91@yahoo.com.mx (E.H.-U.); 2 Department of Pediatric Oncology, National Institute of Pediatrics, Mexico City 04530, Mexico; oncoped_inp@hotmail.com (R.C.-C.);; 3 Department of Oncological Surgery, National Institute of Pediatrics, Mexico City 04530, Mexico; drdanielhdz@gmail.com (D.H.-A.); drjpalacios@hotmail.com (J.P.-A.); drshalkow@gmail.com (J.S.-K.)

**Keywords:** pediatric patients, soft-tissue sarcomas (STSs), cytochrome P450 2W1 (CYP2W1), aberrant expression, therapeutic target, STS subtypes, immunotherapy, prodrug activation, rhabdomyosarcoma, synovial sarcoma

## Abstract

Pediatric soft-tissue sarcomas (STSs) are aggressive malignancies with poor prognoses, particularly in recurrent and metastatic cases. Standard therapies, such as cytotoxic chemotherapy, offer limited survival benefits and carry significant toxicities, underscoring the urgent need for innovative therapeutic approaches. CYP2W1, a tumor-specific monooxygenase enzyme, has emerged as a promising therapeutic target due to its aberrant expression in various cancers. However, its role in pediatric STSs remains poorly understood. This study evaluated CYP2W1 expression in 42 pediatric STS samples across seven histological subtypes using qPCR and Western blot analyses. High CYP2W1 expression was detected in 69% of tumor samples at the mRNA level and in 40.5% at the protein level, compared to absent or negligible expression in matched normal tissues (*p* < 0.001). Synovial sarcoma and rhabdomyosarcoma subtypes exhibited the highest CYP2W1 protein expression, at 70% and 62.5%, respectively. Furthermore, CYP2W1 expression was significantly associated with higher histological grade, advanced tumor stage, and a trend toward reduced overall survival (*p* = 0.082). These findings indicate that CYP2W1 is aberrantly expressed in a subset of pediatric STSs, contributing to tumor aggressiveness and highlighting its potential as a novel therapeutic target for these challenging malignancies.

## 1. Introduction

Pediatric soft-tissue sarcomas (STSs) are a rare and heterogeneous group of aggressive malignancies originating from mesenchymal cells, accounting for approximately 8% of all pediatric cancers [1]. These tumors predominantly affect children and adolescents under 18 years of age and encompass over 70 distinct histological subtypes, with rhabdomyosarcoma, synovial sarcoma, and Ewing sarcoma being the most prevalent [2]. Despite advances in multimodal therapies—including surgery, radiotherapy, and chemotherapy—the prognosis for pediatric STSs remains dismal [3]. While survival rates for localized disease have improved, the 5-year overall survival for advanced or metastatic STSs remains as low as 8% [4]. Moreover, existing chemotherapy is largely palliative and comes with significant toxicities and long-term adverse effects [5], underscoring the critical need for targeted therapies to improve outcomes for these vulnerable patients. The cytochrome P450 (CYP) enzyme superfamily consists of monooxygenases responsible for metabolizing endogenous and exogenous compounds, playing critical roles in physiological and pathological processes [6]. Among its members, CYP2W1 has emerged as a promising area of research due to its tumor-specific expression and aberrant reactivation in various human malignancies [7]. In normal tissues, CYP2W1 is naturally expressed during fetal development but is silenced after birth through methylation [8]. Interestingly, recent studies have documented the reactivation of CYP2W1 expression in different cancers of epithelial origin such as the colon, colorectal, adrenal gland, breast, and hepatocellular carcinoma, with prevalence rates ranging from 30% to 60% [9,10,11,12]. However, the precise molecular mechanisms driving CYP2W1 dysregulation in human cancers remain unclear.

Regardless of its potential significance, this enzyme is still classified as an orphan enzyme due to its largely unknown biological function during development and substrate specificity [7]. Clinically, CYP2W1 upregulation has been associated with tumor aggressiveness, including higher metastatic potential, advanced histological differentiation, and advanced tumor stage, making it a potential biomarker for poor prognosis in colorectal cancer and hepatocellular carcinoma [12,13,14].

Capitalizing on CYP catalytic activity, this cancer-specific CYP enzyme offers novel opportunities for developing selective targeted therapies for cancers expressing this enzyme. In preclinical studies, CYP2W1 demonstrates the ability to selectively bioactivate prodrugs, such as duocarmycin analogs and AQ4N, into highly cytotoxic metabolites within tumor cells [15,16,17], thereby minimizing damage to normal tissues. Moreover, as observed in colon cancer cells, its translocation to the tumor cell surface facilitates its use as a tumor-associated antigen for immunotherapy-based treatments [18,19]. Integrating CYP2W1-prodrug activation with immunotherapy positions CYP2W1 as a key player in precision oncology, providing safer and more effective therapeutic options for cancer patients.

Despite its potential, the CYP2W1 expression profile and clinical significance in pediatric soft-tissue sarcomas (STSs) remain largely underexplored. Preliminary screenings by our group on mesenchymal tumors revealed CYP2W1 overexpression in a small subset of rhabdomyosarcomas, the most common pediatric STS subtype [20]. In contrast, CYP2W1 protein was undetectable in the same patients’ corresponding non-tumoral skeletal muscle and healthy skeletal muscle from independent pediatric controls [20,21]. Motivated by these findings and the therapeutic potential of CYP2W1 in cancer, this study aims to comprehensively investigate CYP2W1 expression across major STS subtypes affecting children and adolescents, evaluating its clinical relevance and prognostic value. To our knowledge, this is the first comprehensive analysis of CYP2W1 in multiple pediatric STS subtypes and clinical subsets to date.

## 2. Patients and Methods

### 2.1. Patient and Tissue Specimens

This study was conducted following approval by the Institutional Ethics and Clinical Research Committees of the National Institute of Pediatrics, Mexico (protocols INP-55/2008 and INP-053/2015). Informed consent for tissue use was obtained from patients or their guardians, with all patient information anonymized. The research adhered to the principles outlined in the Declaration of Helsinki. Between 2010 and 2018, 42 paired primary STS tissue and adjacent normal tissue specimens of pediatric patients (aged ≤ 18 years) with STSs were collected undergoing open biopsy or surgical resection at the Department of Surgery, National Institute of Pediatrics. None of the patients had received chemotherapy, radiotherapy, targeted therapy, or immunotherapy before sample collection. Tissue fragments were immediately preserved in RNA stabilization solution (RNAlater; Ambion, Austin, TX, USA) and stored at −80 °C until analysis.

Diagnosis confirmation of all specimens was performed by expert pathologists. Adjacent normal tissue samples, located >5 cm from the tumor margin, were selected based on the tumor’s location and origin. Clinicopathological data, including patient age, tumor site, sex, tumor grade, and stage, were retrieved from pathology reports and medical records.

### 2.2. Patients and Tumor Characteristics Baseline Demographic and Clinicopathologic Features

A total of 42 pediatric patients (<18 years at diagnosis) with newly diagnosed soft-tissue sarcomas (STSs) were included in the analysis. The baseline demographic and clinicopathologic characteristics are summarized in Table 1. Of the cohort, 52% were male, with a median age at diagnosis of 10.5 years (range: 0–18 years). The median tumor size was 9 cm (range: 0.8–20 cm). Tumor location was categorized into three main anatomical regions: extremities (upper and lower limbs): 27 cases (64.3%); trunk (chest, back, abdominal wall, pelvis): 12 cases (28.6%); and head, neck, and peritoneum (HNP): 3 cases (7.1%).

Tumors were classified according to the World Health Organization (WHO) Classification of Soft Tissue Tumors [22]. The FNCLCC grading system and TNM staging were used to assess tumor differentiation and disease progression [23,24].

Seven histological subtypes of STSs were identified: synovial sarcoma (*n* = 10); rhabdomyosarcoma (*n* = 8); extraosseous Ewing sarcoma (*n* = 6); malignant peripheral nerve sheath tumor (MPNST) (*n* = 5); undifferentiated pleomorphic sarcoma (*n* = 5); mesenchymal chondrosarcoma (*n* = 4); and desmoid-type fibromatosis (*n* = 4).

Tumor grading was performed using the Fédération Nationale des Centres de Lutte Contre le Cancer (FNCLCC) system, which evaluates three key parameters: mitotic index, extent of necrosis, and histological differentiation. Based on these criteria, STS tumors were categorized into grade 1, grade 2, and grade 3 [23]. As per this classification, all rhabdomyosarcoma (RMS) and Ewing sarcoma (ES) cases were assigned Grade 3 to maintain consistency in tumor grading across STS subtypes.

Additionally, TNM staging was performed according to the American Joint Committee on Cancer (AJCC) 7th edition guidelines. The TNM classification assesses: T (size and extent of the primary tumor), N (regional lymph node involvement), and M (presence of distant metastasis). These factors were combined to determine an overall prognostic stage group (I–IV) [23], where stage I indicates localized disease and stage IV reflects advanced disease with distant spread [23].

### 2.3. Total RNA and Protein Extraction

Total RNA and protein were extracted from the 42 pairs of STS tissues and adjacent normal tissues using TRIzol™ (Invitrogen, Gaithersburg, MD, USA), following the manufacturer’s protocol as described in our previous studies [20,21]. Briefly, each tissue sample (50–100 mg) was homogenized in 1 mL of TRIzol using a tissue disrupter, mixed with 200-μL of chloroform, and centrifuged at 13,000 rpm for 15 min to separate the organic and aqueous phases. RNA was retained in the upper aqueous phase, while proteins and DNA were partitioned into the lower phenol phase. The aqueous phase was precipitated with isopropanol, and the RNA pellet was washed with 150-μL of 70% ethanol, air-dried, and dissolved in 20 μL of DEPC-treated water. RNA concentration and purity were determined by the 260/280 absorbance ratio using a NanoDrop ND-2000 spectrophotometer (Thermo Fisher Scientific, Wilmington, DE, USA). RNA integrity was assessed via agarose gel electrophoresis, with visual confirmation of expected bands. The extracted RNA was stored at −70 °C until further analysis.

Total protein was isolated from the organic phase by precipitation with isopropyl alcohol, followed by centrifugation. The resulting protein pellet was washed with 0.3 M guanidine hydrochloride in 95% ethanol, then resuspended in a solution containing 10 mM urea and 10% sodium dodecyl sulfate (SDS) and sonicated until fully solubilized. Total protein concentration was measured using the Lowry method. The extracted protein was stored at −70 °C until further analysis.

### 2.4. Reverse Transcription-PCR, and Real-Time Quantitative PCR (qPCR)

Complementary DNA (cDNA) was synthesized from 2 µg of total RNA extracted from 42 primary STS tissue samples and their corresponding normal tissues. The synthesis was performed using random hexamers and the TaqMan Reverse Transcription Kit (Applied Biosystems, Foster City, CA, USA), following the manufacturer’s instructions.

The CYP2W1 mRNA expression level relative to β-actin mRNA level was measured by RT-qPCR using TaqMan probes. Briefly, CYP2W1 mRNA level was measured by RT-qPCR using commercially available TaqMan probes (Applied Biosystems) for CYP2W1 (Assay ID: Hs00908623_m1), with β-actin (Assay ID: 4333762F) as the reference gene serving as an internal control. Each reaction included 2 µL of cDNA, 1x TaqMan PCR Master Mix, and 100 nM of the TaqMan probe, in a final reaction volume of 20 µL. All RT-PCR reactions were performed in triplicate for each sample and analyzed using the ABI Prism 7700 Sequence Detector (Applied Biosystems, Foster City, CA, USA). The fold change in relative expression levels for CYP2W1 was calculated using the 2^−ΔΔCt^ comparative method [24], with β-actin as the internal control. All samples were analyzed in triplicate and by independent experiments.

### 2.5. Western Blot Analysis of CYP2W1

Western blot assays were conducted following a standard protocol. Briefly, 40 µg of protein extracted from each primary STS and adjacent non-tumor tissue sample was loaded per lane. Proteins of CYP2W1-transfected 293T cells (sc-158417; Santa Cruz Biotechnology, Inc., Dallas, TX, USA) were used as a positive control. Proteins were separated on a 7.5% SDS-PAGE gel and transferred to PVDF membranes (Millipore, Billerica, MA, USA). Nonspecific binding sites were blocked with 5% nonfat dry milk in Tris-buffered saline with 0.5% Tween-20 at room temperature for 1 h. The membranes were then incubated overnight at 4 °C with a commercially available, validated polyclonal anti-CYP2W1 antibody (1:1000; ab113910, Abcam, Cambridge, UK). Antigen–antibody complexes were detected using a horseradish peroxidase-conjugated secondary antibody and an enhanced chemiluminescence detection system (Amersham Life Science, Chalfont St. Giles, Buckinghamshire, UK). Membranes were stripped and re-probed with an anti-β-actin antibody (1:3000; ab115777, Abcam, Cambridge, UK) as a loading control.

### 2.6. Statistical Analysis

The sample size was based on the number of treatment-naïve STS patients treated at our center during the study period. Statistical analyses were performed using SPSS Statistics (v20.0, IBM, Armonk, NY, USA) and GraphPad Prism (v6.0, GraphPad Software, San Diego, CA, USA). Group differences were evaluated using *t*-tests for continuous variables and chi-squared or Fisher’s exact tests for categorical variables, as appropriate. Kaplan–Meier survival curves were used to assess overall survival relative to protein expression, with comparisons made using the log-rank test. A *p*-value < 0.05 was considered statistically significant.

Given their distinct biological behavior and non-metastatic nature, desmoid tumors were excluded from the overall survival analysis to ensure an accurate assessment of prognostic factors in malignant STS cases.

## 3. Results

### 3.1. Baseline Demographic and Clinicopathologic Features

A total of 42 pediatric patients diagnosed with soft-tissue sarcomas (STSs) were included in this study. The baseline demographic and clinicopathologic characteristics are summarized in Table 1. Among the cohort, 52% were male, with a median age at diagnosis of 10.5 years (range: 0–18 years).

The median tumor size was 9 cm (range: 0.8–20 cm), with 64.3% (*n* = 27) of tumors measuring >5 cm at diagnosis. Regional lymph node involvement (N1 stage) was observed in 28.6% (*n* = 12) of cases, indicating a high frequency of locally advanced diseases.

Seven distinct histological subtypes of STSs were identified. The most common subtypes were synovial sarcoma (*n* = 10) and rhabdomyosarcoma (*n* = 8), followed by extraosseous Ewing sarcoma (*n* = 6), malignant peripheral nerve sheath tumor (MPNST) (*n* = 5), undifferentiated pleomorphic sarcoma (*n* = 5), mesenchymal chondrosarcoma (*n* = 4), and desmoid-type fibromatosis (*n* = 4).

Regarding tumor location, cases were categorized into three main anatomical regions: 54.8% (*n* = 23) were located in the extremities (upper and lower limbs), 38.1% (*n* = 16) in the trunk (chest, back, abdominal wall, pelvis), and 7.1% (*n* = 3) in the head, neck, and peritoneum (HNP).

The majority of primary STS tumors in our cohort were classified as Grade 3 (high grade) according to the Fédération Nationale des Centres de Lutte Contre le Cancer (FNCLCC) grading system, accounting for 73.8% (*n* = 31) of cases. Grade 2 (intermediate grade) and Grade 1 (low grade) tumors were less frequent, representing 16.7% (*n* = 7) and 9.5% (*n* = 4) of cases, respectively.

As per the FNCLCC grading system, all rhabdomyosarcoma (RMS) and Ewing sarcoma (ES) cases were classified as Grade 3, consistent with their intrinsically high-grade nature, further reinforcing their aggressive biological behavior.

Regarding TNM staging, tumors were categorized as follows:

- Stage I: Tumors <5 cm, no nodal involvement (N0), no distant metastasis (M0) (*n* = 5, 11.9%);
- Stage II: Tumors >5 cm, no nodal involvement (N0), no distant metastasis (M0) (*n* = 5, 11.9%);
- Stage III: Tumors of any size with regional lymph node involvement (N1), but no distant metastasis (M0) (*n* = 25, 59.5%);
- Stage IV: Tumors of any size, regardless of nodal involvement, with distant metastases (M1) (*n* = 7, 16.6%).

These findings highlight the predominance of advanced disease stages (III and IV), large tumor size, and significant nodal involvement at diagnosis, which aligns with previously reported trends in pediatric STSs. The high proportion of advanced-stage cases underscores the importance of early detection strategies and targeted therapeutic approaches to improve clinical outcomes.

### 3.2. Increased CYP2W1 mRNA Expression in STS Tissues

**Figure 1:**
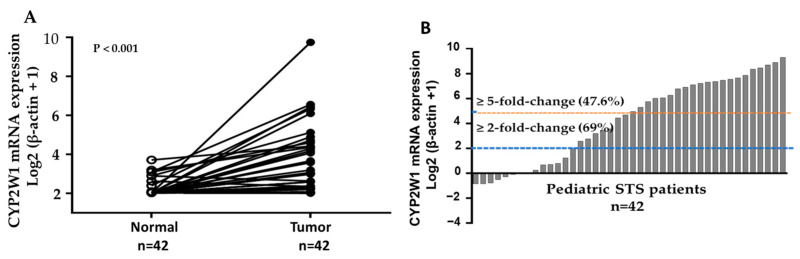
Relative CYP2W1 mRNA expression in pediatric STSs. (**A**) RT-qPCR analysis of relative CYP2W1 mRNA expression levels in 42 paired STS tumor tissues and adjacent normal tissues (*p* < 0.001). β-actin was used as the internal control for normalization. Tumor: STS tumor tissue. Normal: adjacent normal tissue. (**B**) Bar plot depicting CYP2W1 mRNA upregulation in tumor tissues compared to adjacent normal tissues. Upregulation was observed in 29 cases (≥2-fold, blue dashed line) and 20 cases (≥5-fold, red dashed line).

The mRNA level of CYP2W1 was determined by real-time quantitative RT-PCR assays in 42 paired pediatric STS tumor tissues and their corresponding adjacent normal tissues. The expression levels of CYP2W1 mRNA were significantly higher in STS tissues than in matched adjacent normal tissues (*p* < 0.001, Figure 1A). In 29 of 42 cases, CYP2W1 mRNA was upregulated (≥2-fold change) in primary STS tissues relative to adjacent noncancerous tissues. Furthermore, 20 of these cases (47.6%) demonstrated a more pronounced overexpression, with a ≥5-fold increase (Figure 1B). These results showed the upregulation of CYP2W1 mRNA levels in pediatric STSs, suggesting its potential role in tumor biology.

### 3.3. Aberrant Expression of CYP2W1 Protein in STS Tissues

**Figure 2:**
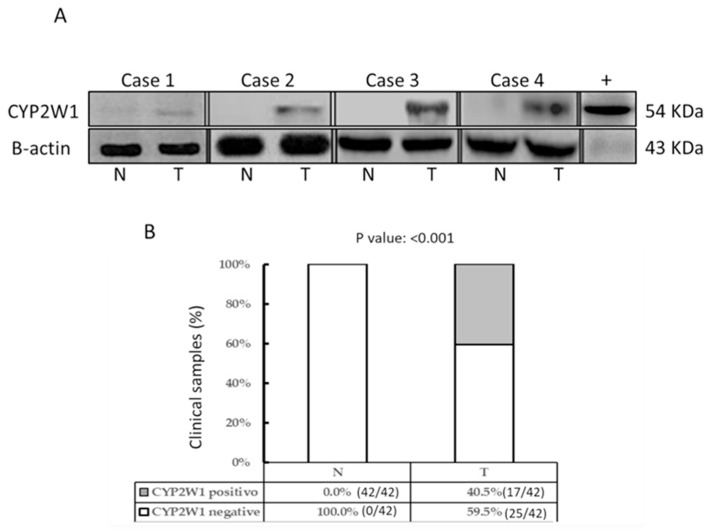
CYP2W1 protein expression in STS tumor tissues. (**A**) Representative Western blot results in STS tumor tissues (T) and adjacent normal tissue (N) across four clinical cases- β-actin was used as a loading control. The positive control is indicated (+). The uncropped blots are shown in the Supplemental Materials. (**B**) Frequency analysis of CYP2W1 protein expression in STS tumor tissues (T) and adjacent normal tissues (N). Aberrant CYP2W1 expression was detected in 40.5% (17/42) of STS tumor tissues, while no expression was observed in normal tissues (0/42). Statistical significance: *p* < 0.001. N: adjacent normal tissues; T: STS tumor tissues; CYP2W1: cytochrome P450 2W1.

CYP2W1 protein expression was analyzed by Western blot in the same tissue samples previously assessed for mRNA levels. Representative immunoblot results are shown in Figure 2A. Consistent with the mRNA expression profile, CYP2W1 protein was overexpressed in a subset of primary STS tumor tissues (17/42, 40.5%), but was undetectable in all paired adjacent normal tissues (0/42) (Figure 2B). Differential expression was significant (*p* < 0.001).

Interestingly, aberrant CYP2W1 protein expression was exclusively observed in STS tumor specimens with significantly elevated mRNA levels (≥5-fold increase compared to normal tissues). This suggests a strong correlation between mRNA abundance and protein translation efficiency, further underscoring the tumor-specific expression of CYP2W1 in pediatric STS tissues.

### 3.4. Frequency of CYP2W1 Protein Expression Across Different Histological STS Subtypes

The frequency of CYP2W1 protein expression showed significant variability across different histological subtypes of pediatric STSs, as summarized in Table 2. Synovial sarcoma exhibited the highest frequency of aberrant CYP2W1 expression, with 70% (7/10) of cases testing positive. Similarly, a high expression rate was observed in rhabdomyosarcoma (RMS), with 62.5% (5/8) of tumors expressing CYP2W1.

Among the eight RMS cases, tumors were further classified into embryonal RMS (ERMS, *n* = 5) and alveolar RMS (ARMS, *n* = 3) based on histopathological and immunohistochemical criteria. CYP2W1 expression was detected in 40% (2/5) of embryonal RMS cases, while 100% (3/3) of alveolar RMS cases tested positive. Although statistical significance could not be achieved due to the limited sample size, CYP2W1 was more frequently expressed in alveolar RMS, a subtype known for its higher metastatic potential and poorer prognosis compared to the embryonal subtype.

In contrast, lower expression frequencies were noted in extraosseous Ewing sarcoma (16.6%, 1/6), malignant peripheral nerve sheath tumor (MPNST) (20%, 1/5), undifferentiated pleomorphic sarcoma (20%, 1/5), and mesenchymal chondrosarcoma (25%, 1/4). Notably, no CYP2W1 expression was detected in desmoid-type fibromatosis (0%, 0/4).

Thus, if these results are validated in large and independent cohorts of pediatric patients, synovial sarcoma and rhabdomyosarcoma—particularly the alveolar subtype—could emerge as the most promising candidates for CYP2W1-based therapeutic strategies, given their notably high expression rates.

### 3.5. Association of Aberrant CYP2W1 Protein Expression with Clinicopathological Features

CYP2W1 protein expression was analyzed in relation to clinicopathological characteristics, grouping cases into CYP2W1-positive (*n* = 17) and CYP2W1-negative (*n* = 25) categories (Table 3). No significant correlation was observed between CYP2W1 expression and patient sex, age, or tumor location (*p* > 0.05). However, CYP2W1 expression was significantly associated with high FNCLCC grade (*p* = 0.043) and advanced TNM stage (*p* = 0.017). The expression was most frequent in Grade 3 (high grade) tumors, observed in 51.6% (16/31) of cases, while it was detected in only 14.3% (1/7) of Grade 2 (intermediate grade) tumors and was absent in Grade 1 (low grade) tumors (0/4). Regarding TNM stages, CYP2W1 expression was observed in 48.0% (12/25) of Stage III STS tumors and 71.4% (5/7) of Stage IV STS tumors, whereas no expression was detected in Stage II or Stage I tumors (0/5 each). These findings highlight a strong correlation between CYP2W1 expression, higher tumor grades, and advanced disease stages (Stages III and IV), suggesting that CYP2W1 may play a crucial role in tumor progression and aggressiveness. This underscores its potential as a biomarker for aggressive pediatric STSs.

### 3.6. Survival Analysis and Prognostic Value of CYP2W1 Protein Expression

Patient survival outcomes were analyzed based on CYP2W1 expression status, considering only malignant STS cases (*n* = 38) after excluding desmoid tumors from the analysis. Patients were grouped into those with negative CYP2W1 expression (49.5%, 25 cases) and those with positive CYP2W1 expression (40.5%, 17 cases).

Kaplan–Meier survival analysis and the log-rank test revealed a trend suggesting an association between CYP2W1 expression and shorter overall survival in pediatric STS patients. However, this trend did not reach statistical significance (log-rank test: *p* = 0.88). Notably, the exclusion of desmoid tumors did not impact the distribution of CYP2W1-positive cases, as all desmoid tumors lacked CYP2W1 expression (0/4 cases). Given the lack of statistical significance, these results are reported in text format rather than as a figure.

These findings underscore the potential role of CYP2W1 in influencing survival outcomes and reinforce the need for further investigation with larger cohorts to confirm its prognostic significance.

## 4. Discussion

Pediatric STSs represent a diverse group of aggressive malignancies with limited systemic treatment options, particularly in advanced or recurrent cases. Current therapies, such as cytotoxic chemotherapy, are associated with significant toxicity and offer only modest survival benefits [5], emphasizing the urgent need for novel, targeted therapeutic approaches to develop more effective and less toxic therapies [25]. In this context, CYP2W1 has emerged as a promising therapeutic target due to its tumor-specific expression and unique metabolic properties [26]. Our findings provide the first comprehensive evidence of aberrant CYP2W1 expression across multiple pediatric STS subtypes and its potential clinical implications.

An initial screening conducted by our group identified CYP2W1 upregulation in a subset of rhabdomyosarcoma tumors, the most common pediatric STS subtype [20]. Building on these findings, this study expands the analysis to characterize CYP2W1 expression across major pediatric STS subtypes, examining its clinicopathological associations and prognostic significance for the first time.

We demonstrated significant upregulation of CYP2W1 at both mRNA and protein levels in pediatric STS tissues compared to negligible or absent expression in adjacent normal tissues. Corresponding CYP2W1 protein was detected in 40.5% of tumor samples, while all matched normal tissues lacked detectable expression, suggesting selective activation of this enzyme in a significant subset of pediatric STSs and reinforcing its potential as a targeted therapeutic candidate. These results align with findings from our initial screening in rhabdomyosarcoma [20]. Importantly, the prevalence of CYP2W1-positive cases and the levels of detectable CYP2W1 observed in our study also are comparable to those reported in some adult epithelial-origin malignancies [26,27,28], where CYP2W1 has shown tumor-selective expression. This consistency suggests that CYP2W1’s tumor-specific reactivation and contribution to malignant behavior may represent a common feature among diverse cancer types, including pediatric STSs.

In the current study, the highest expression frequencies of CYP2W1 protein expression were observed in synovial sarcoma (70%) and rhabdomyosarcoma (62.5%). Among the eight RMS cases analyzed, tumors were further classified into embryonal RMS (*n* = 5) and alveolar RMS (*n* = 3) based on histopathological and immunohistochemical criteria. CYP2W1 expression was detected in 40% (2/5) of embryonal RMS cases and in 100% (3/3) of alveolar RMS cases. Although statistical significance was not reached due to the limited sample size, CYP2W1 was more frequently expressed in alveolar RMS, a subtype known for its higher metastatic potential and poorer prognosis compared to the embryonal subtype. The observed trend, while requiring validation in larger cohorts, suggests a possible link between CYP2W1 expression and the aggressive nature of alveolar RMS.

Other STS subtypes included in this study also displayed aberrant expression but with only one positive case per subtype. In contrast, no CYP2W1 protein expression was detected in desmoid-type fibromatosis, a non-metastatic and well-differentiated subtype [29]. These findings suggest a strong correlation between CYP2W1 expression and tumor aggressiveness, reinforcing its potential role in STS progression. Additionally, they identify synovial sarcoma and rhabdomyosarcoma—particularly alveolar RMS—as key subtypes that could benefit from CYP2W1-targeted therapies in the future due to the frequent expression of CYP2W1 in these aggressive tumors. However, the study’s limited sample size and uneven subtype representation restrict definitive conclusions. Larger, more balanced cohorts are needed to validate these results and further clarify CYP2W1’s role across specific STS subtypes.

Significant associations were determined between CYP2W1 protein expression and histological grade and stage of the disease, both recognized markers of tumor progression [30]. No CYP2W1 expression was observed in low-grade (Grade 1) or early-stage (Stage I, II) STS tumors, while high-grade (Grade 3) and advanced-stage (Stages III and IV) cases showed prominent expression. Specifically, CYP2W1 was detected in 51.6% of high-grade tumors and 71.4% of Stage IV cases, suggesting a potential role for CYP2W1 in promoting tumor aggressiveness and progression in pediatric STSs. These findings align with prior studies reporting that CYP2W1 expression is associated with more aggressive tumor phenotypes and advanced disease stages in other malignancies. The lack of expression in low-grade and early-stage STS tumors (Stage I, II) further emphasizes its potential as a marker of disease severity.

To ensure a clinically meaningful survival analysis, desmoid tumors were excluded due to their borderline nature, non-metastatic behavior, and complete absence of CYP2W1 expression. The revised analysis, now focused solely on malignant STS cases (*n* = 38), demonstrated a non-significant trend (*p* = 0.88), suggesting an association between CYP2W1 expression and reduced survival. While this observation aligns with findings in other aggressive tumor subtypes, larger studies are necessary to confirm this relationship.

Additionally, TNM staging was selected to maintain consistency across all STS subtypes. The TNM system provided a standardized framework for evaluating tumor progression, ensuring comparability between different histological types. Furthermore, no significant correlation was found between CYP2W1 expression and patient sex, age, or tumor location (*p* > 0.05), reinforcing that CYP2W1 expression is more closely linked to tumor biology rather than demographic factors.

Moreover, survival analysis revealed a trend toward shorter overall survival in patients with CYP2W1-positive tumors, although this did not reach statistical significance (*p* = 0.088). This observation is consistent with prior studies in colorectal and hepatic carcinomas, where CYP2W1 has been linked to tumor aggressiveness and poor prognosis [27,28]. The potential role of CYP2W1 as a prognostic biomarker in pediatric STSs highlights its relevance in predicting outcomes; however, larger cohorts with extended follow-up are required to validate these trends and establish the independent prognostic value of CYP2W1 in this population.

To date, CYP2W1 stands out as the most tumor-specific member of the cytochrome P450 family identified in STS tumors. Unlike CYP1B1 and CYP2E1—two other notable P450 enzymes that, while expressed in various cancers, including pediatric STSs [31], are also present in corresponding normal tissues—CYP2W1 appears to be exclusively upregulated in tumor tissues, making it a uniquely selective therapeutic target. However, our study did not investigate the molecular mechanisms driving CYP2W1 activation in tumor tissues. Prior studies suggest that epigenetic dysregulation, such as CpG island demethylation, may play a critical role in reactivating CYP2W1 expression [8,9]. Similar epigenetic mechanisms are known to regulate other oncofetal genes, including carcinoembryonic antigen and trophoblast glycoprotein, which are aberrantly re-expressed in cancer [32]. Therefore, future research is required to clarify the molecular mechanisms of CYP2W1 reactivation in primary STS tissues

The fact that CYP2W1 is aberrantly overexpressed in a significant subset of primary STS tissues but is found absent in normal tissues makes it a promising therapeutic target for the treatment of cancers expressing CYP2W1. These therapeutic strategies are CYP2W1-based immunotherapy and CYP2W1-directed prodrugs. CYP2W1-based therapies could selectively target tumor cells, providing safer and more effective treatment options, especially for aggressive subtypes like synovial sarcoma and rhabdomyosarcoma. CYP2W1’s ability to bioactivate prodrugs, such as duocarmycin analogs and AQ4N, into cytotoxic agents highlights its substantial therapeutic potential [26]. Furthermore, antibody-drug conjugates targeting CYP2W1 offer innovative treatment strategies that could enhance precision and efficacy [19]. While CYP2W1-specific therapies are not yet commercially available, these approaches hold great promise for improving outcomes in pediatric STSs. The development of CYP2W1-focused therapies, including prodrugs and immunotherapy, is essential to address the unmet clinical needs of this aggressive cancer.

Although our study findings are promising, we acknowledge some limitations that should be addressed in the future. First, due to the rarity of pediatric STSs and the single-center nature of this study, the sample size and imbalance of histological subtypes included in our research were limited, and hence, the study was subject to selection bias. Second, our study cohort included a relatively small number of patients, and the follow-up time for evaluating patient survival was relatively short, which caused less statistical power. Third, the underlying mechanisms of CYP2W1 in pediatric STS patients were not investigated in this study. Therefore, our observations require large-scale clinical trials and multicenter clinical studies to be confirmed, involving more pediatrics together with defined larger groups of each subtype, as well as clarifying the molecular mechanisms of CYP2W1 overexpression in STSs.

## 5. Conclusions

In summary, our research demonstrates that CYP2W1 is aberrantly expressed in a significant subset of primary STS tumors, with the highest prevalence rates in synovial sarcoma and rhabdomyosarcoma. Among RMS subtypes, CYP2W1 expression was more frequently observed in alveolar RMS, reinforcing its potential association with a more aggressive clinical course. Additionally, CYP2W1 expression was significantly associated with an advanced phenotype, including high FNCLCC grade and TNM stage III-IV. These findings suggest that CYP2W1 could serve as a promising therapeutic target; however, its utility would be limited to individuals whose tumors demonstrate CYP2W1 expression. Future research should focus on validating its clinical relevance and exploring personalized therapeutic approaches tailored to CYP2W1-positive pediatric STS cases, offering a more targeted and effective treatment strategy.

## Figures and Tables

**Table 1:** Summary of patient characteristics and clinicopathological features (*n* = 42).

Clinical Data	Number of Cases (%)
Age (years) Median (range) ≤ 10 >10	10.5 (0–18) 15 (35.7) 27 (64.3)
Tumor size (cm) Median (range)	9 cm (0.8–20.0)
Sex Male Female	22 (52.4) 20 (47.6)
Tumor location Extremities Trunk Head, neck, and peritoneum	23 (54.8) 16 (38.1) 3 (7.1)
Histological subtype Synovial sarcoma Rhabdomyosarcoma Extraosseous Ewing sarcoma Malignant peripheral nerve sheath tumor (MPNST) Undifferentiated pleomorphic sarcoma (UPS) Mesenchymal chondrosarcoma Desmoid-type fibromatosis	10 (23.8) 8 (19.0) 6 (14.3) 5 (11.9) 5 (11.9) 4 (9.5) 4 (9.5)
FNCCLCC grade G1 G2 G3	4 (9.5) 7 (16.7) 31 (73.8)
TNM stage Stage I: tumors ≤ 5 cm, N0, M0 Stage II: tumors > 5 cm, N0, M0 Stage III: tumors of any size, N1, M0 Stage IV: tumors of any size, N0 or N1, M1	5 (11.9) 5 (11.9) 25 (59.5) 7 (16.6)

FNCLCC grade: Fédération Nationale des Centres de Lutte Contre le Cancer, Sarcoma Group grading system. TNM staging: Tumor-Node-Metastasis classification system. N0: no nodal involvement; N1: regional Lymph node involvement; M0: no distant metastasis; M1: distant metastasis.

**Table 2:** Frequency of CYP2W1 protein expression across histological STS subtypes.

Histological Subtypes	Total of Cases (*n*)	Positive Cases (*n*)	Percentage Positive (%)
Synovial sarcoma	10	7	70.0
Rhabdomyosarcoma (RMS)	8	6	62.5
Embrional RMS (ERMS)	5	2	40.0
Alveolar RMS (ARMS)	3	3	100
Ewing sarcoma	6	1	16.7
MPNST	5	1	20.0
UPS	5	1	20.0
Mesenchymal chondrosarcoma	4	1	25.0
Desmoid-type fibromatosis	4	0	0

CYP2W1: Cytochrome P450 2W1; STS: Soft Tissue Sarcoma; MPNST: Malignant Peripherial Nerve Sheat Tumor; UPS: Undifferentiated Pleomorphic Sarcoma.

**Table 3:** Relationship between CYP2W1 protein expresión with clinicopathological features in pediatric STS.

		Protein Expression		
Clinical Data	Number (*n* = 42)	Positive (*n* = 17)	Negative (*n* = 25)	*p*-Value
Age (years) ≤10 ≥10	15 27	9 8	6 19	0.183
Sex Male Female	22 20	12 5	10 10	0.065
Tumor location Extremities Trunk Head, neck and peritoneum	23 16 3	13 4 0	10 12 3	0.141
FNCLCC grade G1 G2 G3	4 7 31	0 1 16	4 6 15	0.043 *
TNM stage I II III IV	5 5 27 7	0 0 12 5	5 5 13 2	0.017 *

FNCLCC grade: Fédération Nationale des Centres de Lutte Contre le Cancer system; TNM stage: Tumor-Node-Metastasis clasiffication system; CYP2W1: Cytocrome P450 2W1. * *p* < 0.05.

## Data Availability

The data presented in this study are available on request from the corresponding author. The data are not publicly available due to privacy and ethical concerns.

## Supplementary Materials

The following supporting information can be downloaded at: https://www.mdpi.com/article/10.3390/curroncol32030131/s1, Figure S1. Full Length Western Blot Images Corresponding to Figure 2A (N, adjacent normal tissue; T: STS tumor tissue).
