# Supplementary figures and images for: Aberrant Expression of CYP2W1 in Pediatric Soft Tissue Sarcomas: Clinical Significance and Potential as a Therapeutic Target
Source: Curr Oncol. 2025 Feb 26;32(3):131. doi: 10.3390/curroncol32030131 (PMC11941694; doi:10.3390/curroncol32030131)

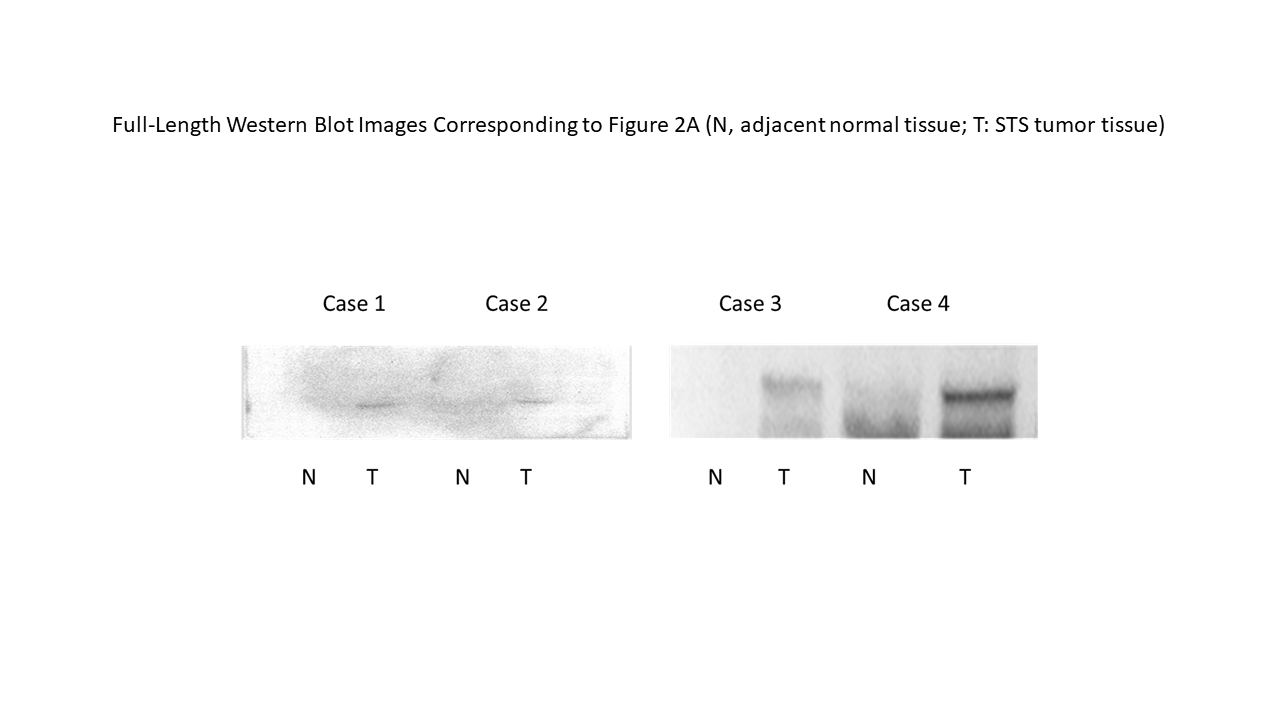

Supplement: Supplementary file 1 [file curroncol-32-00131-s001.zip › Figure S1.tif]
